# Imbibition and Oil Drainage Mechanisms of Nanoparticle Compound Polymer Fracturing Fluids

**DOI:** 10.3390/gels12020136

**Published:** 2026-02-02

**Authors:** Herui Fan, Tianyu Jiang, Ruoxia Li, Yu Si, Yunbo Dong, Mingwei Zhao, Zhongzheng Xu, Lin Li

**Affiliations:** 1 State Key Laboratory of Deep Oil and Gas, China University of Petroleum (East China), Qingdao 266580, China; herui8503@163.com (H.F.); tianyujiang_upc@163.com (T.J.); upc_lrx@163.com (R.L.); yusi_frank@163.com (Y.S.); 15590262179@163.com (Y.D.); zhaomingwei@upc.edu.cn (M.Z.); 2 School of Petroleum Engineering, China University of Petroleum (East China), Qingdao 266580, China

**Keywords:** nanoparticle, linear gel fracturing fluid, slickwater, imbibition mechanism

## Abstract

Unconventional low-permeability reservoirs present significant production challenges due to the poor imbibition and displacement efficiency of conventional polymer fracturing fluids. The injection of nanoparticle (NP) compounds into polymer fracturing fluid base systems, such as linear gels or slickwater, has garnered significant research interest due to their superior performance. However, previous studies have primarily focused on evaluating the fluid’s properties, while its imbibition and oil displacement mechanisms within reservoirs remain unclear. Herein, the imbibition mechanism of nanoparticle composite polymer fracturing fluid was systematically investigated from macro and micro perspectives using low-field nuclear magnetic resonance (LF-NMR), atomic force microscopy (AFM), interfacial rheology, and other technical means. The results showed that the imbibition recovery using polymer fracturing fluid was 10.91% higher than that achieved with conventional slickwater. Small and medium pores were identified as the primary contributors to oil drainage. Nanoparticles can be adsorbed on the rock wall in the deep reservoir to realize wettability reversal from oil-wet to water-wet, reducing crude oil adhesion. Furthermore, a strong interaction between the adsorbed NPs and cleanup agents at the oil–water interface was observed, which reduces interfacial tension to 0.95 mN·m^−1^, mitigates the Jamin effect, and enhances interfacial film deformability. NPs increase the interfacial dilatational modulus from 6.0 to 14.4 mN·m^−1^, accelerating fluid exchange and oil stripping. This work provides a consolidated mechanistic framework linking NP-induced interfacial modifications to enhanced pore-scale drainage, offering a scientific basis for designing next-generation fracturing fluids. We conclude that NP-compound systems hold strong potential for low-permeability reservoir development, and future efforts must focus on optimizing NP parameters for specific reservoir conditions and overcoming scalability challenges for field deployment.

## 1. Introduction

With the continuous development of oilfields, the production from conventional oilfields is becoming increasingly difficult, and the contradiction between the supply and demand of oil is becoming increasingly serious [1,2,3]. At present, unconventional oil and gas reservoirs are widely distributed and abundant [4,5,6]. Therefore, the focus of oil and gas exploration and development has gradually shifted from conventional to unconventional reservoirs, which is an inevitable trend in the development of the petroleum industry [7,8,9]. To economically and efficiently develop unconventional oil and gas resources, especially low-permeability reservoirs, hydraulic fracturing technology is often used to enhance oil recovery [10,11,12]. Fracturing fluid, as the medium for transferring pressure during hydraulic fracturing, has functions such as transporting high pressure, generating and enlarging fractures, and carrying proppants. Its performance determines the effectiveness of hydraulic fracturing and the level of oil and gas production [13,14,15,16]. Polymer fracturing fluids (such as linear gels or slickwater) are widely used in the development of low-permeability reservoirs, due to their low operating cost and low formation damage [17,18,19,20]. The core components of both slickwater (a low-viscosity linear gel) and linear gel fracturing fluids are polyacrylamide-based polymer, which exhibit low interfacial activity and poor imbibition performance [21,22].

To further improve the oil recovery, researchers have considered improving the imbibition performance of slickwater fracturing fluid through new materials. Since the late 1980s, when “nanocrystals” were first artificially prepared, research on nanomaterials has been introduced into various industries [23,24,25]. Particularly, researchers in the field of oil and gas are paying increasing attention to nanomaterials [26,27,28,29]. Currently, nanomaterials are widely used in areas such as water injection and drilling fluid additives within the petroleum industry. For example, the injection of 0.2% nano-polysilicon (SiO_2_) into low-permeability oil reservoirs can reverse wettability and ultimately lower injection pressure, increasing water-phase permeability by approximately 49% [30]. The addition of specific nanoparticles to drilling fluids can form a high-strength filter cake, significantly enhancing wellbore stability. Adding 0.5 wt% functionalized multi-walled carbon nanotubes (FMWCNTs) to drilling fluids reduces filtration loss by up to 66.66% [31]. Due to their high surface activity, large specific surface area, and strong selective adsorption, NPs can not only reduce the interfacial tension between oil and water but also change the wettability of the rock surface to improve the oil recovery efficiency in reservoirs. Studies have found that CTAB-MoS_2_ nanofluids can reduce oil–water interfacial tension from 36 mN/m to 14.9 mN/m; polymer-coated SiO_2_ nanoparticles can change the rock contact angle from 143.3° to 48.75°, achieving oil-wet to water-wet alteration; a silica nanofluid emulsion (1 wt%) can reduce water injection pressure by more than 40% [32]. The introduction of suitable nanoparticles into slickwater or linear gels can enhance their imbibition performance and further achieve the synergistic enhancement of fracturing, imbibition, and oil displacement. However, previous research reports mainly focused on evaluating the performance of nanomaterial-compound fracturing fluids, but fewer studies have addressed the mechanism behind the enhanced performance [33,34].

To clarify the imbibition and drainage mechanism of nanoparticle compound fracturing fluids in reservoirs, this paper systematically investigates the fluid–oil–rock interaction mechanism from both macroscopic and microscopic perspectives. This is achieved through imbibition and drainage experiments combined with techniques such as low-field nuclear magnetic resonance (NMR), atomic force microscopy (AFM), and interfacial rheology. The research results are of great significance for the efficient development of unconventional oil and gas reservoirs.

## 2. Results and Discussion

### 2.1. Influence of Liquid Type on the Imbibition Recovery

**Figure 1:**
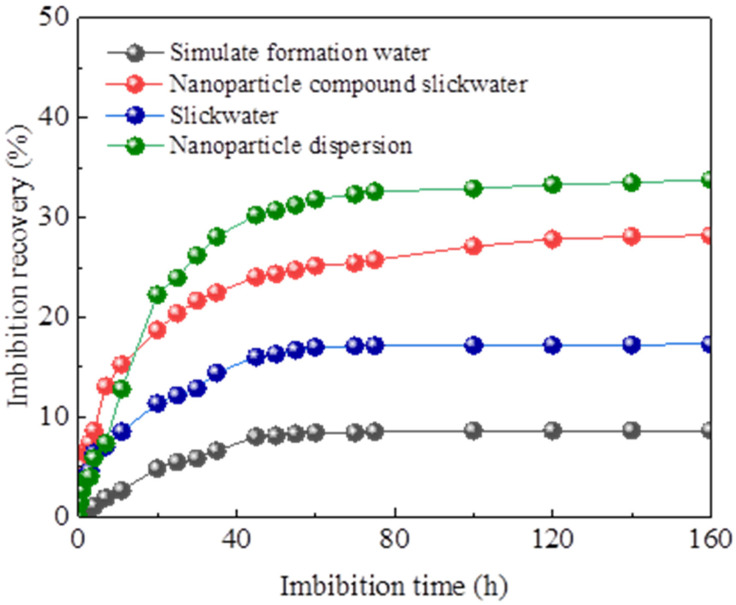
Spontaneous imbibition recovery curve of the core in different imbibition liquids.

As illustrated in Figure 1, the recovery of different imbibition fluids followed the descending order: nanoparticle dispersion > nanoparticle compound slickwater > slickwater without nanoparticles > simulated formation water. During the initial imbibition stage, both the oil drainage rates of nanoparticle dispersion and nanoparticle compound slickwater exhibited significantly higher oil displacement rates than the slickwater without nanoparticles. This enhancement is attributed to the ability of nanoparticles to penetrate deep into the reservoir, adsorb onto the rock surface, and alter its wettability [35,36]. This process reduces the adhesion work, thereby facilitating the mobilization and drainage of crude oil droplets from the core. The imbibition recovery of the nanoparticle compound slickwater increased by 19.57% and 10.91% compared with the simulated formation water and slickwater separately. The results demonstrate that after the incorporation of nanoparticles, the imbibition performance of the compound slickwater was enhanced.

### 2.2. Characteristics of Pore Throat During Imbibition and Oil Drainage Process

According to previous research, there was a linear correspondence between lateral relaxation time and core pore radius, as shown in Equation (1) [37]:

*r* = *CT*_2_ (1)

where *C* is the conversion coefficient, μm·ms^−1^; *T*_2_ is the relaxation time, ms; *r* is the pore radius, μm.

**Figure 2:**
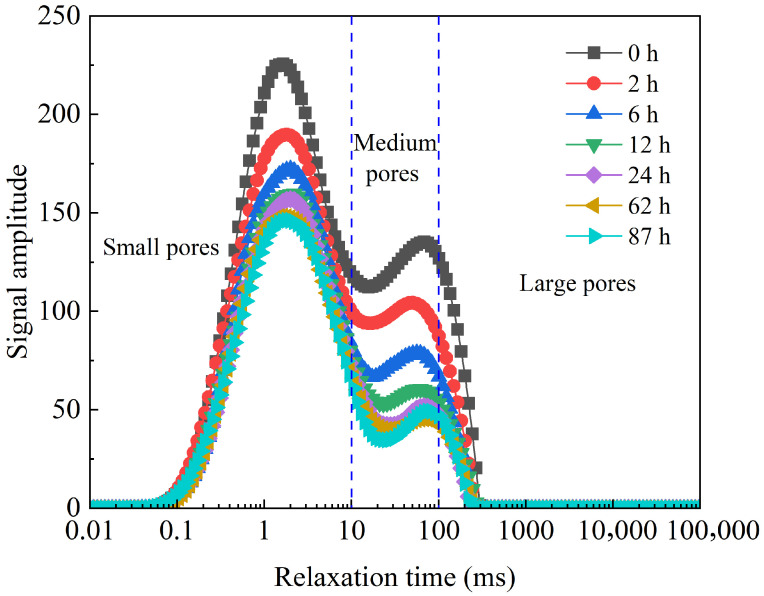
NMR *T*_2_ spectrum of the imbibition and oil drainage process of nanoparticle compound slickwater at different times.

The core pores could be divided into three types, small pores, medium pores, and large pores, according to the *T*_2_ thresholds established in the reference [38]. Pores with a relaxation time of less than 10 ms were classified as small pores, pores with a relaxation time in the range of 10 ms~100 ms were classified as medium pores, and pores with a relaxation time of more than 100 ms were classified as large pores. For the nanoparticle compound slickwater, the *T*_2_ spectra of the imbibition process at different times of 2 h, 6 h, 12 h, 24 h, 62 h, and 87 h are shown in Figure 2.

**Figure 3:**
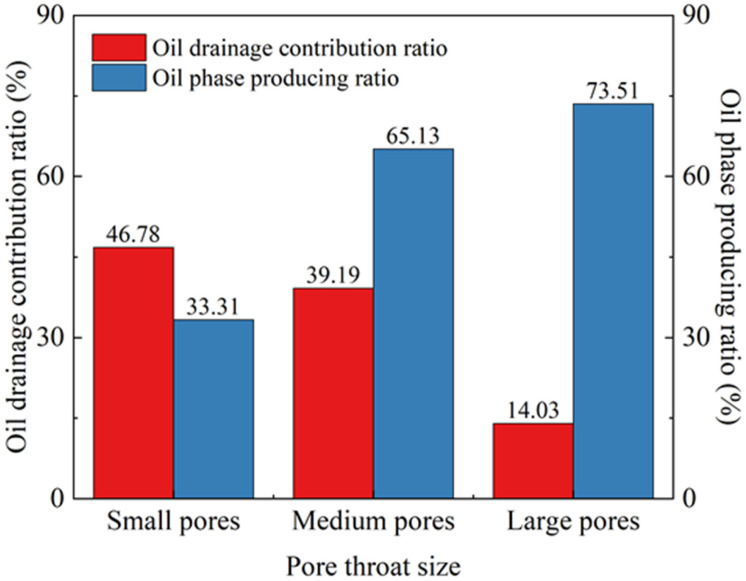
Oil drainage contribution ratio and oil phase producing ratio of different pores.

As shown in Figure 2, at the initial state (0 h) of imbibition, the amplitude of the nuclear magnetic resonance signal was strongest. Crude oil was distributed to varying degrees in small, medium, and large pores. After 2 h of imbibition, the crude oil in the core was rapidly drained. In the early stage (<12 h), the amplitude of the *T*_2_ spectrum signal decreased rapidly, while in the middle stage (>12 h), it decreased slowly. In the late stage (>24 h), the rate of decrease in signal amplitude became gradual, which aligned with the previous results from the imbibition and oil drainage experiments (Figure 1). After the imbibition time reached 87 h, the peak area for large pores increased, indicating that the remaining oil within the large pores had increased. This is likely attributable to the aggregation of small oil droplets drained by imbibition from the small and medium pores during this stage. Subsequently, these small oil droplets flowed into the large pores, which have lower flow resistance, resulting in a slight macroscopic increase in the remaining oil within large pores. Based on the low-field nuclear magnetic resonance *T*_2_ peak areas of the nanoparticle compound slickwater, the oil drainage contribution ratio and the oil phase producing ratio for pores of different scales could be calculated. As shown in Figure 3, the oil drainage contribution ratios (i.e., the ratio of the imbibition peak area of pores with different scales to that of the whole core at the end of the process) for small pores and medium pores were 46.78% and 39.19%, respectively, which were significantly higher than those for large pores (14.03%). Therefore, small pores and medium pores were the main contributing regions for oil drainage by the nanoparticle compound slickwater.

The contribution to crude oil recovery was defined as the ratio of the oil drainage area of pores with different scales at the end of the process to the area of the initial oil-saturated oil. The recovery contribution degree of crude oil from small pores was relatively low (33.31%), whereas the contributions from medium and large pores were higher (65.13% and 73.51%, respectively). During the imbibition process, the nanoparticle compound slickwater migrated along pore throats toward the core interior under capillary action, displacing the remaining oil. Due to the difference in diameter of the internal pores of the core, there was a capillary force difference between the larger pores and the smaller pores, allowing imbibition to occur spontaneously. In general, the smaller the reservoir pore size, the greater the capillary force (imbibition force) formed. However, the complex pore structure often led to poor connectivity between pores, resulting in a threshold pressure for residual oil mobilization [39]. During the process, nanoparticles adsorb onto the rock surfaces, facilitating the stripping of residual oil. When the pore size was smaller than a certain value, the ability of nanoparticles to strip oil became limited. Furthermore, the capillary force may be insufficient to overcome the starting pressure of residual oil, preventing the nanoparticle compound slickwater from entering the smallest pores. Consequently, the contribution degree of crude oil recovery of the small pores was the lowest among the three types of pores.

By analyzing the initial saturated oil state and the peak area changes in the *T*_2_ spectrum of the core after a certain period of imbibition, the imbibition recovery during the imbibition process could be calculated, as shown in Equation (2):

(2) $$R=\frac{S_{0}-S_{i}}{S_{0}}\times 100\%$$

where *S*_0_ is the peak area of the *T*_2_ spectrum in the initial saturated oil state; *S_i_* is the peak area of the *T*_2_ spectrum after a certain period of imbibition and drainage; *R* is the imbibition recovery.

**Figure 4:**
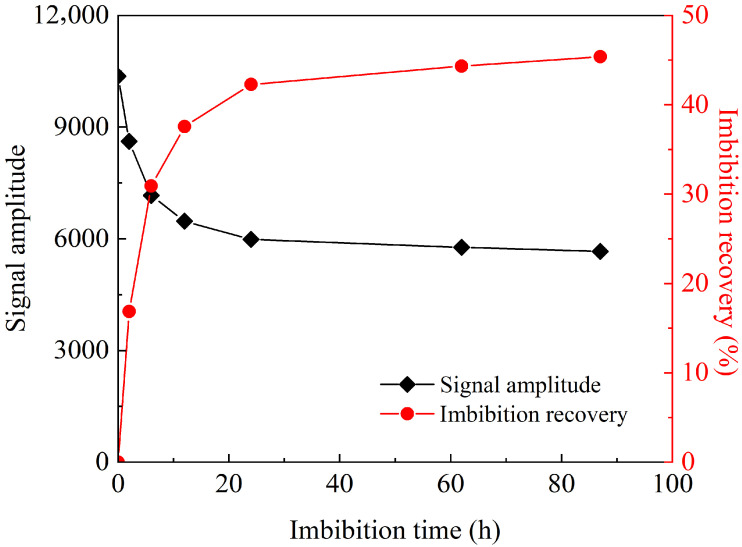
*T*_2_ peak area and imbibition recovery of nanoparticle compound slickwater in the imbibition process.

According to the *T*_2_ peak area of the nanoparticle compound slickwater at different imbibition times, the dynamic variation in imbibition recovery over time during the process was obtained. As shown in Figure 4, for the nanoparticle compound slickwater, the imbibition recovery at 2 h, 6 h, 12 h, 24 h, 62 h, and 87 h was 14.46%, 28.16%, 35.07%, 39.44%, 41.79%, and 42.85%, respectively. The initial imbibition rate was high. As imbibition progressed, the imbibition rate gradually slowed down until the recovery nearly stabilized. In the NMR experiments, a small amount of simulated oil drained by imbibition adhered to the wall of the imbibition bottle, resulting in less oil being recorded than was displaced. Thus, the imbibition recovery calculated from the low-field NMR *T*_2_ spectrum peak area was higher than that measured in the imbibition experiments (Figure 1).

**Figure 5:**
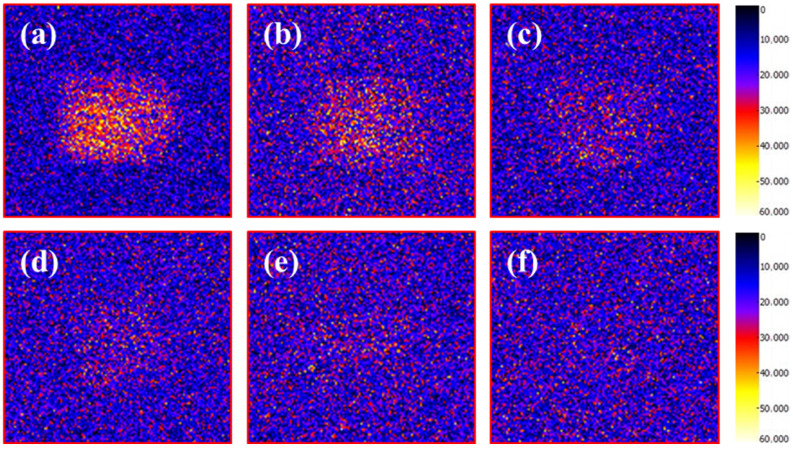
Images of the simulated oil distribution at different imbibition times: (**a**) 0 h; (**b**) 2 h; (**c**) 6 h; (**d**) 12 h; (**e**) 24 h; (**f**) 62 h.

When imaging cores using low-field NMR technology, a higher oil saturation within the pores results in a stronger hydrogen nucleus signal amplitude, leading to a brighter area in the corresponding image. Cores treated with nanoparticle compound slickwater for different imbibition durations were imaged, and these images were pseudo-colored using a Hot Metal mode. It could be seen from Figure 5 that at 0 h of imbibition (i.e., the initial state of imbibition), the color of the core area was bright, and the rectangular boundary contour was clear. After 2 h of imbibition, the core area was darkened rapidly, and its boundary contour began to blur. The nanoparticle compound slickwater rapidly replaced the oil phase inside the core to the outside under the action of imbibition. The amplitude of the hydrogen nuclear signal corresponding to the oil was significantly reduced. By 6 h, the core area was further darkened, indicating that the system had replaced the remaining oil from deeper within the core. Compared with the previous stage, the imbibition rate was slightly reduced. At 12 h, the oil-phase signal in the core area was only marginally higher than the background level, indicating that the imbibition rate was stabilizing. After imbibition for more than 24 h, the oil phase signal remained virtually unchanged. During this late stage, the imbibition rate was extremely low, and no significant increase in recovery was observed for the nanoparticle compound slickwater.

### 2.3. Core Surface Adsorption Characteristics After the Fracturing Fluid Imbibition

**Figure 6:**
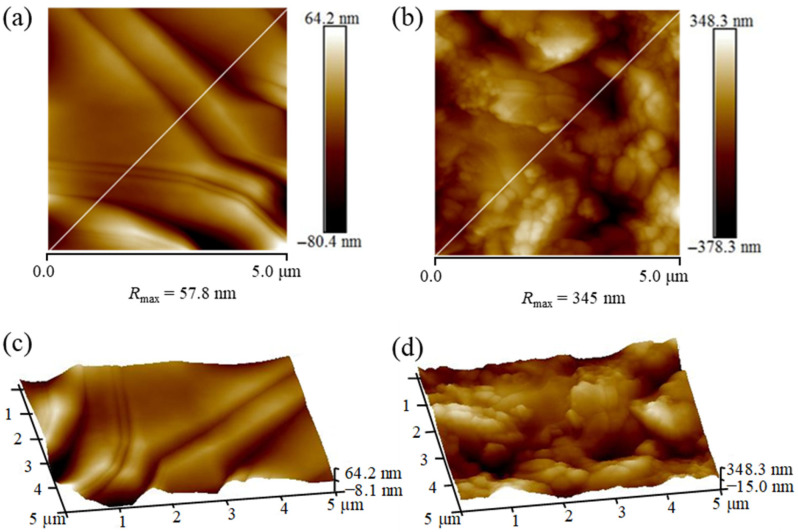
Surface adsorption characteristics of cores treated with different imbibition fluids: (**a**) and (**b**) show the maximum roughness of cores treated with slickwater and nanoparticle compound slickwater, respectively; (**c**) and (**d**) represent the 3D morphology of core slices treated with slickwater and nanoparticle compound slickwater, respectively.

The adsorption morphologies of slickwater without nanoparticles and nanoparticle compound slickwater on the core surface were characterized by AFM. It can be seen from Figure 6a that the core treated with slickwater without nanoparticles was relatively smooth, with almost no solid components being adsorbed on the surface. The maximum difference in surface roughness R_max_ measured by AFM was 57.8 nm. In contrast, Figure 6b shows that the core treated with nanoparticle compound slickwater formed a densely packed granular area due to the adsorption of nanoparticles on the surface, with the maximum difference in surface roughness R_max_ of 345 nm. Combined with the 3D morphology images in Figure 6c,d, compared with slickwater without nanoparticles, many nanoparticles were tightly adsorbed on the surface when the core was treated with the nanoparticle compound slickwater. A dense adsorption layer had been formed. It indicated that nanoparticles could change the roughness of the core surface and reduce the adhesion work of crude oil, further stripping off the oil in the core [40]. In summary, as the system gradually migrated towards the deeper part of the reservoir, more residual oil within the pores of the reservoir could be drained.

### 2.4. Wettability Reversal of the Core Surface After Fracturing Fluid Imbibition

**Figure 7:**
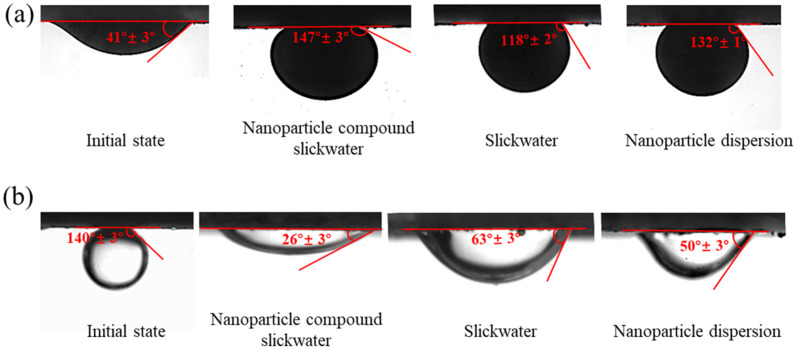
Changes in wetting angle before and after treatment with different systems: (**a**) oil-phase wetting angle; (**b**) water-phase wetting angle.

Figure 7a shows the oil contact angles for different systems on the core surface. The initial oil contact angle of the core was 41°, and the surface of the core was oleophilic. After separate treatment by the nanoparticle compound slickwater, slickwater without nanoparticles, and nanoparticle dispersion, the wettability of the core surface changed significantly. As shown in Figure 7a, the oil contact angles increased to 147°, 118°, and 132°, respectively. Compared with other systems, the nanoparticle compound slickwater contained more adsorbable components. There was a synergistic effect between nanoparticles and cleanup agent molecules, which could form a dense compound adsorption layer on the core surface, making the core surface more capable of wettability reversal. Compared with the cleanup agent, nanoparticles could adsorb at the oil–water–solid three-phase contact interface, forming a wedge-shaped structure separation pressure, and making the oil phase wetting angle of the nanoparticle dispersion greater than that of the slickwater without nanoparticles. Thus, the crude oil on the core surface was more easily stripped off. As shown in Figure 7b, there were differences in the water phase contact angles of different systems on the core surface. The initial water phase contact angle of the core was 140°, and the surface of the core was oleophilic. After separate treatment by the nanoparticle compound slickwater, slickwater without nanoparticles, and nanoparticle dispersion, the water phase contact angles decreased to 26°, 63°, and 50°, respectively. Therefore, based on the ability of wettability reversal, the order was as follows: nanoparticle compound slickwater > nanoparticle dispersion > slickwater without nanoparticles. The experimental results were consistent with the previous oil phase contact angle measurement results. Nanoparticles and cleanup agent molecules in compound slickwater can both migrate to the deep part of the reservoir and form a dense adsorption layer on the core surface, causing wetting reversal of the core surface. The original oil-wet core surface was reversed to a water-wet surface. The adhesion work of oil droplets was reduced, making it easier to be stripped off from the wall. Therefore, the imbibition and oil drainage ability of compound slickwater was enhanced.

### 2.5. Effect of Oil–Water Interfacial Tension on Imbibition Recovery

**Figure 8:**
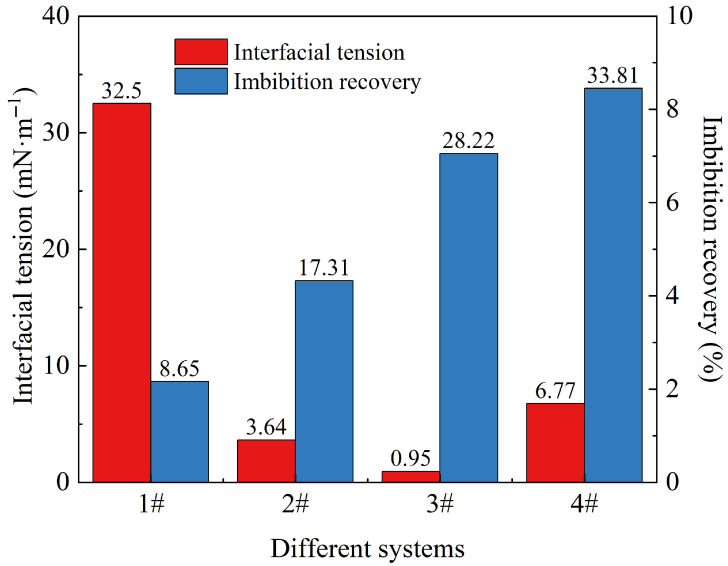
Oil–water interfacial tension and imbibition recovery of different systems: 1# is the simulated formation water, 2# is slickwater without nanoparticles, 3# is nanoparticle compound slickwater, and 4# is nanoparticle dispersion.

Based on the previously measured imbibition recovery of different systems, the relationship between interfacial tensions and imbibition recovery was further analyzed. The results are shown in Figure 8. The interfacial tension values for the simulated formation water, slickwater without nanoparticles, nanoparticle compound slickwater, and nanoparticle dispersion were 32.50 mN·m^−1^, 3.64 mN·m^−1^, 0.95 mN·m^−1^, and 6.77 mN·m^−1^, respectively, with corresponding imbibition recoveries of 8.65%, 17.31%, 28.22%, and 33.81%. Except for simulated formation water, the other three systems significantly reduced the oil–water interfacial tension. The interfacial tension of nanoparticle compound slickwater was the lowest (0.95 mN·m^−1^). A synergistic effect between nanoparticles and cleanup agent molecules in this system could further reduce the interfacial tension. Particles and cleanup agent molecules absorbed at the oil–water interface, reducing the adhesion work of oil droplets. Furthermore, nanoparticles adsorbed on the core surface could form a wedge-shaped separation pressure, facilitating the stripping of oil droplets [41]. Additionally, a low interfacial tension can mitigate the Jamin effect, resulting in a decrease in the seepage resistance of the oil droplets [42]. Consequently, the imbibition and oil drainage capacity of the nanoparticle compound slickwater was further enhanced.

### 2.6. Oil–Water Interfacial Behavior of Different Imbibition Liquids

Interface rheological properties were the main indicators used to describe the adsorption behavior of various components on the interface. When the interface undergoes periodic contraction or expansion, the interface tension also undergoes periodic changes. Among them, the value of the interfacial expansion modulus was equal to the ratio of the change in surface tension to the change in interface area, as shown in Equation (3) [43]:

(3) $$E=\frac{d\gamma }{dA/A}=\frac{d\gamma }{d\ln A}$$

where *γ* represents the interfacial tension, mN·m^−1^; *A* is the interface area, m^2^; *E* denotes the interfacial expansion modulus, mN·m^−1^.

**Figure 9:**
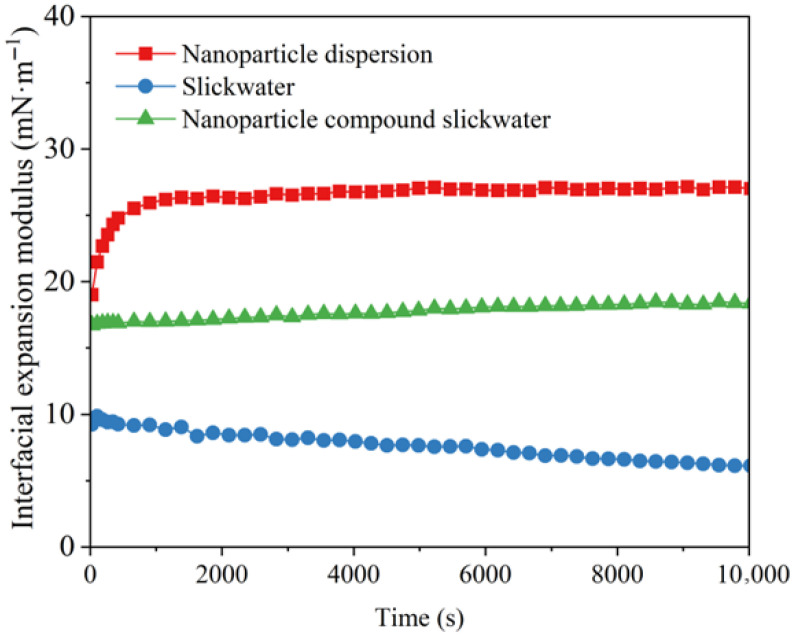
Interfacial expansion modulus curves of different systems with time.

By comparing the interfacial expansion moduli of different systems, their adsorption behavior at the oil–water interface can be analyzed. A higher expansion modulus of the oil–water interface promotes faster diffusion and exchange between the bulk phase and the interface, thereby enhancing the deformation capability of oil droplets [44]. As shown in Figure 9, the interfacial expansion moduli follow the descending order: nanoparticle dispersion > nanoparticle compound slickwater > slickwater, which were 27.0 mN·m^−1^, 14.4 mN·m^−1^, and 6.0 mN·m^−1^. As the nanoparticle dispersion continuously migrates deeper into the reservoir, nanoparticles adsorb at the oil–water interface, strengthening the interfacial film and causing a rapid increase in the expansion modulus. When the adsorption of nanoparticles on the interface reaches saturation, the interfacial expansion modulus stabilizes. While in the slickwater without nanoparticles, the relaxation process of polymer drag-reducer molecules at the oil–water interface was slow, and the interfacial expansion modulus gradually decreased. In the nanoparticle compound slickwater, there was a strong interaction between nanoparticles and cleanup agent molecules, and the diffusion exchange rate between the bulk phase and the interface was accelerated. This makes the oil–water interfacial film more deformable, thereby enhancing the mobility of oil droplets and facilitating the displacement of residual oil from the core. The mechanistic understanding developed herein provides a valuable framework for the design of nano-enhanced fracturing fluids. However, a critical assessment reveals significant challenges that must be navigated for successful field deployment. Beyond the laboratory, the complex heterogeneity, mineralogy, and fluid chemistry of real formations may substantially modify the nanoparticle transport and interfacial interactions observed in idealized core systems.

## 3. Conclusions

This study establishes a clear mechanism linking nanoscale interfacial activity to enhanced oil recovery in low-permeability reservoirs using nanoparticle compound polymer fracturing fluid. Core findings show that small and medium pores are the primary contributors to oil displacement, accounting for 85.97% of total recovery. Mechanistically, NPs enhance recovery through synergistic dual-interface action: they reverse rock wettability to reduce oil adhesion while simultaneously lowering interfacial tension to 0.95 mN·m^−1^ and increasing the interfacial dilatational modulus to 14.4 mN·m^−1^ at the oil–water interface, thereby promoting droplet mobilization. The rapid increase signifies an accelerated diffusion-exchange rate between the bulk phase and the interface, as well as an enhanced interfacial film strength. In addition, the adsorption of nanoparticles renders the oil–water interface film more deformable, which improves the mobility of oil droplets. In summary, nanoparticles optimize the pore-scale force balance by co-modifying rock–fluid and oil–water interfaces, providing a validated framework for designing high-performance fracturing fluids through interfacial engineering. The nanoparticle compound slickwater, with its superior imbibition capability, can significantly improve post-fracturing oil recovery and demonstrates excellent potential for the development of low-permeability oil and gas reservoirs.

## 4. Experimental Section

### 4.1. Experimental Materials

The drag reducer FZ60 (≥90.0%) was supplied by Beijing Xitao Technology Development Co., Ltd., Beijing, China. Nanoparticles with a surface containing hydroxyl and sulfonic groups (SF-101, ≥90.0%) were self-made in the laboratory, and particle size of SF-101 was less than 20 nm [45]. The cleanup agent (NP-6, ≥90.0%) was provided by Guangzhou Nanjia Chemical Co., Ltd., Guangzhou, China. The anti-swelling agent potassium chloride (KCl, ≥99.7%), sodium bicarbonate (NaHCO_3_, ≥99.7%), sodium chloride (NaCl, ≥99.7%), magnesium chloride hexahydrate (MgCl_2_·6H_2_O, ≥99.7%), manganese (II) chloride tetrahydrate (MnCl_2_·4H_2_O, ≥99.7%), and calcium chloride (CaCl_2_, ≥99.7%) were all provided by China Sinopharm Chemical Reagent Co., Ltd., Shanghai, China. Ultrapure water was self-made in the laboratory. Dodecane (98%) was provided by China Shanghai Macklin Biochemical Technology Co., Ltd., Shanghai, China. The simulated oil was prepared by mixing dehydrated crude oil with kerosene at a volume ratio of 1:3, resulting in a density of 0.832 g·cm^−3^ and a viscosity of 2.55 mPa·s. The simulated formation water had a total dissolved solids (TDS) content of 30,048.9 mg·L^−1^; its detailed ion composition is listed in Table 1. The artificial cores were provided by Beijing Tiandi Kaiyuan Geological Technology Co., Ltd., Beijing, China, and their specific parameters are presented in Table 2.

### 4.2. Compositions of the Imbibition Liquids

(1) Slickwater fracturing fluid compositions: 0.05 wt% FZ60 (drag reducer) + 0.05 wt% NP-6 (cleanup agent) + 0.7 wt% KCl (anti-swelling agent) + water.
(2) Nanoparticle compound fracturing fluid: 0.05 wt% FZ60 (drag reducer) + 0.05 wt% SF-101 (nanoparticles) + 0.05 wt% NP-6 (cleanup agent) + 0.7 wt% KCl (anti-swelling agent) + water. The functional performances of this fracturing fluid system for field applications—including its wellbore friction reduction capability, temperature and shear resistance, and reservoir damage characteristics—have been thoroughly evaluated in our previous work [45].
(3) Nanoparticle dispersion solution: 0.05 wt% SF-101 (nanoparticles) + water.

### 4.3. Core Imbibition Experiment

The influence of different fluids on imbibition recovery was evaluated through imbibition and drainage experiments. The specific procedures were as follows: First, the dry weight of each core was measured. Subsequently, the core was saturated with simulated oil using the vacuum-pressure saturation method and then aged for 7 days at the reservoir temperature (80 °C) to establish equilibrium wettability. Following this, its wet weight was measured. Next, the core was immersed in imbibition bottles containing imbibition fluids, and the bottles were placed in a constant-temperature water bath maintained at 80 °C. The volume of simulated oil drained from the core was recorded at regular time intervals to calculate the imbibition recovery. This study primarily employed the volumetric method to conduct the imbibition experiments. By recording the volume of oil drained at various time points, the curve of imbibition recovery over time was calculated using Equation (4) [46]:

(4) $$E=\frac{\rho V}{m_{1}-m_{0}}\times 100\%$$

where *ρ* is the simulated oil density, g·cm^−3^; *V* is the drained oil volume, mL; *m*_0_ is the dry weight of the core, g; *m*_1_ is the wet weight of the core, g; *E* is the imbibition recovery of the core, %.

### 4.4. Pore-Throat Characteristics of Cores in the Process of Imbibition

Low-field nuclear magnetic resonance (NMR) technology was employed to investigate changes in pore-throat characteristics during the imbibition process of the nanoparticle compound slickwater into reservoir cores. The experimental procedure was as follows. First, the dry weight of the core was measured. The core was then saturated with simulated oil using the vacuum-pressure saturation method and aged at the reservoir temperature (80 °C) for 7 days, after which its wet weight was measured. A nanoparticle-enhanced slickwater (used as the imbibition fluid) was prepared using a 20% MnCl_2_ solution as the base fluid. This system is used to shield the hydrogen nucleus signal of the aqueous phase in the imbibition fluid. Therefore, the detected changes in the transverse relaxation time (*T*_2_) spectrum curve only reflect the changes in the hydrogen nucleus signal of the oil phase in the experimental core. This is a standard practice in this technique [37]. The core was immersed in a bottle containing imbibition liquid. The imbibition bottle was placed in a constant temperature water bath at 80 °C, and the core was taken out at a certain time interval. *T*_2_ spectrum measurements and imaging analysis were conducted using an online low-field nuclear magnetic resonance analysis system. Following each measurement, the core was returned to the imbibition fluid to await the next interval.

### 4.5. Surface Adsorption Characteristics of the Core After Imbibition

The core was sectioned into two small cylindrical disks with a diameter of 1 cm, and the end faces of the two core slices were polished using sandpaper. Subsequently, the slices were cleaned ultrasonically and dried in an oven at 80 °C for 48 h. Each disk was then immersed separately for 12 h: one in conventional slickwater (without nanoparticles) and the other in nanoparticle-enhanced slickwater. Atomic force microscopy (AFM) was employed to measure the microscopic morphology of each component in the different systems after adsorption on the core slice surface. The scanning area set by the probe on the core slice was 5 μm × 5 μm. Finally, the adsorption characteristics of the core surfaces were investigated and analyzed.

### 4.6. Wettability Measurement of Cores After Imbibition

Multiple core slices with comparable properties were saturated with crude oil and aged for 7 days. These slices were then immersed separately for 24 h in three different fluids: nanoparticle compound slickwater, slickwater without nanoparticles, and nanoparticle. The changes in both the core surface wetting angle of the oil phase and the water phase were measured using a contact angle measuring instrument (JC2000D, Shanghai Zhongchen Digital Technology Co., Ltd., Shanghai, China) via the rising drop method at ambient conditions (25 °C, 1 atm). The volume of each droplet was fixed at 2.0 μL. For each sample, at least five measurements on different locations were averaged. For these measurements, the water phase was laboratory-prepared ultrapure water, and the oil phase was the simulated oil.

### 4.7. Measurements of the Oil–Water Interfacial Tension and Expansion Modulus

The changes in oil–water interfacial tension of four simulated systems, namely formation water, slickwater without nanoparticles, nanoparticle compound slickwater, and nanoparticle dispersion, were studied using the rotary droplet interfacial tension meter (TX500C) (Shanghai Zhongchen Digital Technology Co., Ltd., Shanghai, China). The experiments were conducted at 80 °C with a rotational speed of 6000 r·min^−1^. The expansion modulus of the oil–water interface between the slickwater without nanoparticles, nanoparticle compound slickwater, nanoparticle dispersion, and simulated oil was measured using an interface rheometer (Tracker) (TECLIS Scientific, Civrieux d’Azergues, France). The measurement method was the droplet shape method. The experimental temperature was 25 °C, and the oscillation mode was area oscillation, with an amplitude of 10% of the oil droplet area and a frequency of 0.1 Hz.

## Figures and Tables

**Table 1:** Ionic composition of simulated formation water.

Ionic Type	Na^+^	K^+^	Ca^2+^	Mg^2+^	Cl^−^	HCO_3_^−^	SO_4_^2−^
Concentration/(mg·L^−1^)	9060.0	524.5	26.0	12.2	6274.7	13,959.4	192.1

**Table 2:** Core parameters.

Core Number	Length/cm	Diameter/cm	Porosity/%	Permeability/(×10^−3^ μm^2^)
1#	2.56	2.54	15.12	1.70
2#	2.33	2.52	16.42	1.71
3#	2.41	2.52	15.83	1.71
4#	2.41	2.50	15.82	1.73
5#	2.52	2.50	15.32	1.76

## Data Availability

The original contributions presented in the study are included in the article, further inquiries can be directed to the corresponding authors.
